# Dichlorido{(*E*)-4-dimethyl­amino-*N*′-[(pyri­din-2-yl)methyl­idene-κ*N*]benzo­hydrazide-κ*O*}zinc

**DOI:** 10.1107/S1600536812049355

**Published:** 2012-12-08

**Authors:** Manuel N. Chaur

**Affiliations:** a Departamento de Química, Facultad de Ciencias, Universidad del Valle, AA 25360, Santiago de Cali, Colombia

## Abstract

In the mononuclear title complex, [ZnCl_2_(C_15_H_16_N_4_O)], the Zn^II^ cation is five-coordinated in a strongly distorted square-pyramidal environment by two Cl^−^ anions and a neutral tridentate Schiff base ligand. The Zn^II^ cation is chelated by the carbonyl O atom, the imine N atom and the pyridine N atom, which causes a slight loss of planarity for the ligand; the dihedral angle between the aromatic rings is 4.61 (8)°.

## Related literature

For related structures, see: Moreno-Fuquen *et al.* (2012 ▶); Chaur *et al.* (2011 ▶); Ma *et al.* (2011 ▶). For the structure of the ligand and its complex with CuCl_2_, see: Sangeetha, Pal & Pal (2000 ▶); Sangeetha, Pal, Anson *et al.* (2000 ▶). For the design of mol­ecular dynamic systems, see: Hirose (2010 ▶); Lehn (2006 ▶). For the synthetic principles of compounds exhibiting dynamic properties, see Kay *et al.* (2007 ▶). For information storage, see: Kandel (2001 ▶).
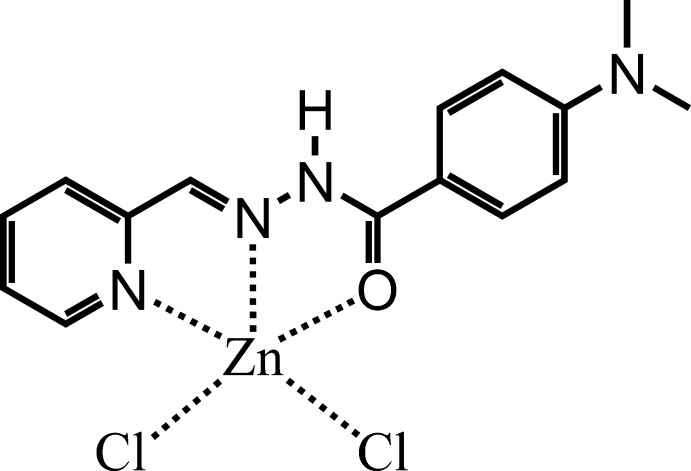


## Experimental

### 

#### Crystal data

[ZnCl_2_(C_15_H_16_N_4_O)]
*M* *_r_* = 404.59
Monoclinic, 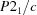

*a* = 16.1822 (7) Å
*b* = 13.5864 (7) Å
*c* = 7.5989 (2) Å
β = 91.123 (3)°
*V* = 1670.36 (12) Å^3^
*Z* = 4
Mo *K*α radiation
μ = 1.80 mm^−1^
*T* = 173 K
0.40 × 0.22 × 0.10 mm

#### Data collection

Nonius KappaCCD diffractometer
Absorption correction: multi-scan (MULscanABS in *PLATON*; Spek, 2009 ▶) *T*_min_ = 0.538, *T*_max_ = 0.764
14366 measured reflections
3803 independent reflections
3252 reflections with *I* > 2σ(*I*)
*R*_int_ = 0.055

#### Refinement

*R*[*F* ^2^ > 2σ(*F* ^2^)] = 0.035
*wR*(*F* ^2^) = 0.084
*S* = 1.06
3803 reflections
210 parameters
H-atom parameters constrained
Δρ_max_ = 0.33 e Å^−3^
Δρ_min_ = −0.74 e Å^−3^

### 

Data collection: *COLLECT* (Nonius, 1998 ▶); cell refinement: *DENZO* (Otwinowski & Minor, 1997 ▶); data reduction: *DENZO*; program(s) used to solve structure: *SHELXS97* (Sheldrick, 2008 ▶); program(s) used to refine structure: *SHELXL97* (Sheldrick, 2008 ▶); molecular graphics: *PLATON* (Spek, 2009 ▶); software used to prepare material for publication: *SHELXL97*.

## Supplementary Material

Crystal structure: contains datablock(s) I, global_Publ_Block. DOI: 10.1107/S1600536812049355/bh2455sup1.cif

Structure factors: contains datablock(s) I. DOI: 10.1107/S1600536812049355/bh2455Isup2.hkl

Additional supplementary materials:  crystallographic information; 3D view; checkCIF report

## Figures and Tables

**Fig. 1.**
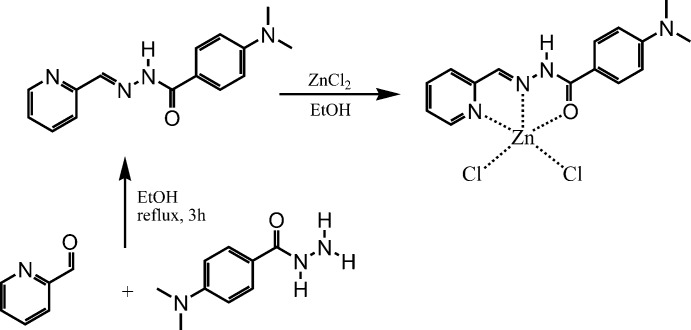
The synthetic route for the title complex.

**Fig. 2.**
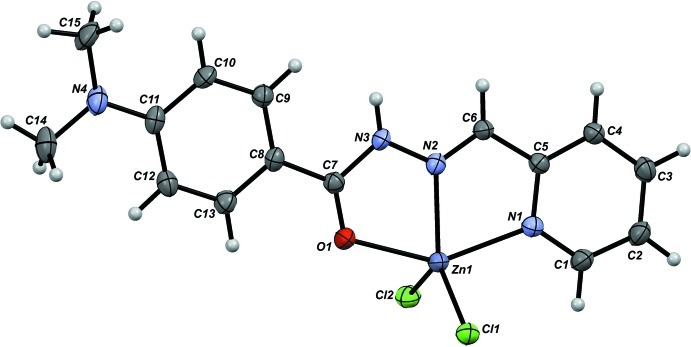
The structure of the title compound with displacement ellipsoids drawn at the 50% probability level.

**Fig. 3.**
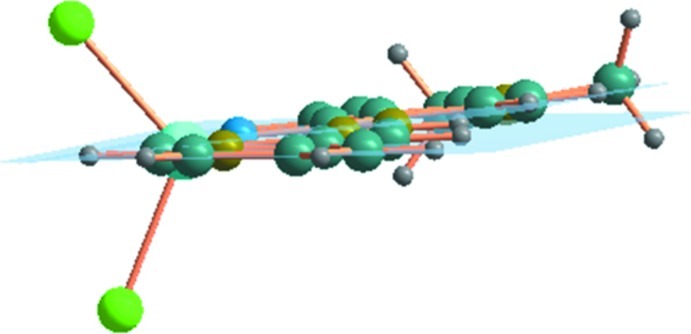
Molecular structure of the title compound showing in light blue the dihedral angle formed between the aromatic and pyridyl rings.

**Fig. 4.**
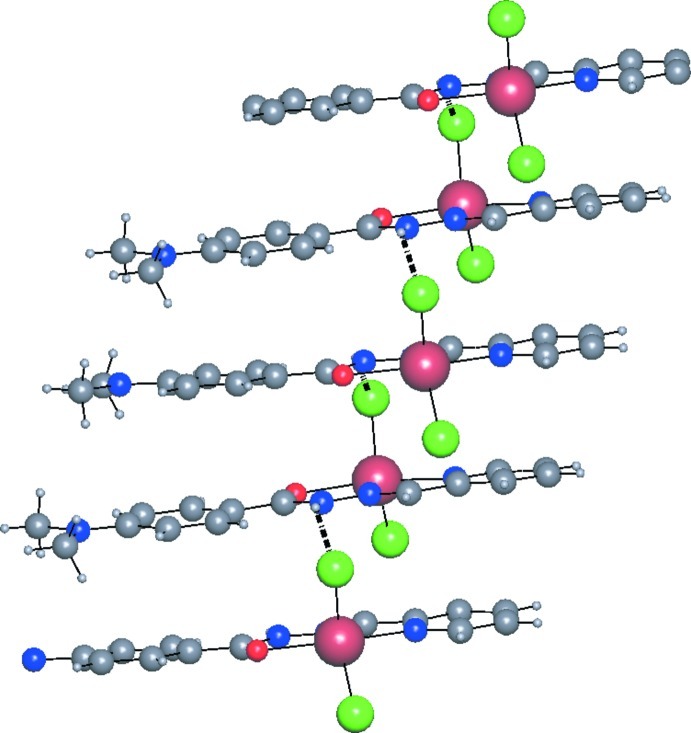
Stacking of the molecules forming a column along the [001] direction.

## supplementary crystallographic information

### Comment

Similar to bis-pyridyl hydrazones derivatives, pyridine-2-carboxaldehyde acyl (aroyl) hydrazones are able to undergo configurational (*E*/*Z*) isomerization and constitutional changes as well as their structure allows them to coordinate to metallic centers by a tridentade NNO binding site (Chaur *et al.*, 2011). Therefore, the C=N bond of these hydrazones can be used in double dynamic processes of interest for information storage (Lehn, 2006; Kay *et al.*, 2007). The configurational dynamics of these compounds give access to short term photoactivated metastable states. On the other hand, they can undergo constitutional dynamics by constituent exchange allowing long term storage of information (Kandel, 2001).

Thus the pyridyl-acyl hydrazones are appealing compounds for the design of systems exhibiting multiple states and interconversion processes that involve configurational/constitutional changes, as well as metal coordination (Ma *et al.*, 2011). These features together with increasing time scales being of interest for the development of both short-term and long-term molecular information storage and processing devices that may be addressed by orthogonal transformations involving either physical stimuli (light, heat; see Hirose, 2010) or chemical effectors (amino components or metal cations).

In this regard our group focuses on the design of bis-pyridyl and pyridyl-acyl hydrazones, as the title compound (Fig. 1), for the implementation of dynamic systems exhibiting reversible multiplex states for information storage (Moreno-Fuquen *et al.*, 2012). The new complex is based on a ligand for which the structure has been previously established (Sangeetha, Pal & Pal, 2000), as well as a Cu(II) complex (Sangeetha, Pal, Anson *et al.*, 2000).

The title complex exhibits a distorted five-coordinated square-pyramidal disposition (Fig. 2). The Schiff base ligand is not planar (Fig. 3), resulting in a dihedral angle between the planes of the aromatic and pyridyl rings of 4.61 (8)°, while the free ligand is planar (Sangeetha, Pal & Pal, 2000). The molecules stack forming columns along the [001] direction by intermolecular hydrogen bonds with a distance N3—H3···Cl2 = 3.199 (2) Å. Also it is observed a weak π–π slipped stacking interaction between the aromatic rings, with separations between ring centroids of 3.8075 (1) Å (Fig. 4).

### Experimental

(*E*)-4-(Dimethylamino)-*N*'-(pyridin-2-ylmethylene)benzohydrazide: 2-pyridinecarboxaldehyde (1.0 equivalent) was added to an ethanol solution of 4-(dimethylamino)benzohydrazine (1.0 equiv.) and a trace amount of glacial acetic acid. The reaction mixture was refluxed for three hours, then the precipitate was collected in a Büchner funnel and recrystallized from ethanol affording the ligand in a 90% yield.

[Zn(C_15_H_16_N_4_O)Cl_2_]: Two hot ethanolic solutions of the previously prepared Schiff base ligand and ZnCl_2_ in stoichiometric proportions were mixed and then allowed to cool. The complex salt crystallized out. Then the product was recrystallized from ethanol. Crystals suitable for X-ray diffraction were obtained by slow diffusion of methanol over a DMSO solution of the zinc complex.

### Refinement

All H atoms were placed in idealized positions, with C—H bond lengths fixed to 0.93 (aromatic CH) or 0.96 Å (methyl), and refined as riding with displacement parameters calculated as *U*_iso_(H) = *xU*_eq_(carrier C) where *x* = 1.2 (aromatic CH) or 1.5 (methyl).

### Crystal data

**Table:**

[ZnCl_2_(C_15_H_16_N_4_O)]	*F*(000) = 824
*M**_r_* = 404.59	*D*_x_ = 1.609 Mg m^−^^3^
Monoclinic, *P*2_1_/*c*	Mo *K*α radiation, λ = 0.71073 Å
Hall symbol: -P 2ybc	Cell parameters from 18477 reflections
*a* = 16.1822 (7) Å	θ = 1.0–27.5°
*b* = 13.5864 (7) Å	µ = 1.80 mm^−^^1^
*c* = 7.5989 (2) Å	*T* = 173 K
β = 91.123 (3)°	Plate, orange
*V* = 1670.36 (12) Å^3^	0.40 × 0.22 × 0.10 mm
*Z* = 4	

### Data collection

**Table:**

Nonius KappaCCD diffractometer	3803 independent reflections
Radiation source: fine-focus sealed tube	3252 reflections with *I* > 2σ(*I*)
Graphite monochromator	*R*_int_ = 0.055
φ and ω scans	θ_max_ = 27.5°, θ_min_ = 1.3°
Absorption correction: multi-scan (MULscanABS in *PLATON*; Spek, 2009)	*h* = −18→20
*T*_min_ = 0.538, *T*_max_ = 0.764	*k* = −17→16
14366 measured reflections	*l* = −9→8

### Refinement

**Table:**

Refinement on *F*^2^	Primary atom site location: structure-invariant direct methods
Least-squares matrix: full	Secondary atom site location: difference Fourier map
*R*[*F*^2^ > 2σ(*F*^2^)] = 0.035	Hydrogen site location: inferred from neighbouring sites
*wR*(*F*^2^) = 0.084	H-atom parameters constrained
*S* = 1.06	*w* = 1/[σ^2^(*F*_o_^2^) + (0.0401*P*)^2^ + 0.7957*P*] where *P* = (*F*_o_^2^ + 2*F*_c_^2^)/3
3803 reflections	(Δ/σ)_max_ = 0.001
210 parameters	Δρ_max_ = 0.33 e Å^−^^3^
0 restraints	Δρ_min_ = −0.74 e Å^−^^3^
0 constraints	

### Fractional atomic coordinates and isotropic or equivalent isotropic displacement parameters (Å^2^)

**Table:**

	*x*	*y*	*z*	*U*_iso_*/*U*_eq_	
C1	0.00605 (14)	0.89834 (18)	0.1655 (3)	0.0286 (5)	
H1A	0.0063	0.9667	0.1708	0.034*	
C2	−0.06416 (14)	0.85138 (19)	0.0983 (3)	0.0307 (5)	
H2A	−0.1101	0.8875	0.0608	0.037*	
C3	−0.06401 (14)	0.75050 (19)	0.0887 (3)	0.0291 (5)	
H3A	−0.1098	0.7172	0.0428	0.035*	
C4	0.00513 (13)	0.69839 (17)	0.1480 (3)	0.0241 (5)	
H4A	0.0064	0.6300	0.1422	0.029*	
C5	0.07173 (12)	0.75049 (16)	0.2157 (3)	0.0206 (4)	
C6	0.14641 (13)	0.70244 (16)	0.2877 (3)	0.0223 (4)	
H6A	0.1522	0.6344	0.2903	0.027*	
C7	0.32992 (13)	0.79835 (16)	0.4744 (3)	0.0219 (4)	
C8	0.40823 (13)	0.76645 (17)	0.5528 (3)	0.0226 (4)	
C9	0.42999 (13)	0.66764 (18)	0.5805 (3)	0.0250 (5)	
H9A	0.3925	0.6183	0.5502	0.030*	
C10	0.50558 (14)	0.64279 (19)	0.6515 (3)	0.0284 (5)	
H10A	0.5182	0.5768	0.6697	0.034*	
C11	0.56503 (13)	0.71553 (19)	0.6978 (3)	0.0256 (5)	
C12	0.54238 (14)	0.81434 (19)	0.6719 (3)	0.0282 (5)	
H12A	0.5794	0.8640	0.7032	0.034*	
C13	0.46628 (13)	0.83866 (18)	0.6010 (3)	0.0270 (5)	
H13A	0.4530	0.9047	0.5845	0.032*	
C14	0.70184 (15)	0.7651 (2)	0.8079 (3)	0.0408 (7)	
H14A	0.7102	0.8067	0.7077	0.061*	
H14B	0.7531	0.7341	0.8414	0.061*	
H14C	0.6825	0.8040	0.9042	0.061*	
C15	0.65998 (16)	0.5884 (2)	0.8059 (4)	0.0396 (6)	
H15A	0.6212	0.5644	0.8897	0.059*	
H15B	0.7149	0.5843	0.8552	0.059*	
H15C	0.6564	0.5492	0.7009	0.059*	
N1	0.07305 (11)	0.84965 (14)	0.2226 (2)	0.0232 (4)	
N2	0.20292 (10)	0.76034 (14)	0.3464 (2)	0.0216 (4)	
N3	0.27499 (11)	0.72631 (14)	0.4181 (2)	0.0232 (4)	
H3B	0.2857	0.6645	0.4278	0.028*	
N4	0.64094 (12)	0.69027 (16)	0.7633 (3)	0.0328 (5)	
O1	0.31050 (10)	0.88558 (12)	0.4524 (2)	0.0295 (4)	
Cl1	0.13487 (3)	1.01282 (4)	0.52926 (7)	0.02717 (13)	
Cl2	0.23435 (3)	0.98562 (4)	0.07806 (7)	0.02852 (14)	
Zn1	0.189086 (15)	0.914569 (19)	0.32708 (3)	0.02243 (9)	

### Atomic displacement parameters (Å^2^)

**Table:**

	*U*^11^	*U*^22^	*U*^33^	*U*^12^	*U*^13^	*U*^23^
C1	0.0269 (12)	0.0245 (13)	0.0343 (12)	0.0053 (9)	−0.0051 (9)	−0.0028 (9)
C2	0.0244 (12)	0.0315 (14)	0.0360 (12)	0.0074 (10)	−0.0066 (9)	−0.0026 (10)
C3	0.0229 (11)	0.0323 (14)	0.0317 (11)	−0.0025 (10)	−0.0045 (9)	−0.0051 (10)
C4	0.0247 (11)	0.0212 (12)	0.0263 (10)	−0.0005 (9)	−0.0021 (8)	−0.0015 (9)
C5	0.0212 (10)	0.0209 (11)	0.0196 (9)	−0.0010 (9)	0.0001 (8)	−0.0004 (8)
C6	0.0223 (11)	0.0193 (11)	0.0252 (10)	0.0011 (9)	−0.0027 (8)	−0.0010 (8)
C7	0.0212 (10)	0.0224 (12)	0.0221 (10)	−0.0005 (9)	0.0006 (8)	−0.0015 (8)
C8	0.0179 (10)	0.0283 (12)	0.0216 (10)	0.0019 (9)	0.0004 (8)	−0.0007 (9)
C9	0.0214 (11)	0.0264 (12)	0.0271 (10)	−0.0007 (9)	−0.0012 (8)	0.0005 (9)
C10	0.0245 (12)	0.0259 (13)	0.0345 (12)	0.0054 (9)	−0.0036 (9)	0.0031 (10)
C11	0.0181 (10)	0.0376 (14)	0.0210 (10)	0.0018 (10)	−0.0001 (8)	0.0021 (9)
C12	0.0218 (11)	0.0338 (13)	0.0290 (11)	−0.0046 (10)	−0.0028 (9)	0.0015 (10)
C13	0.0237 (11)	0.0275 (13)	0.0297 (11)	0.0023 (10)	−0.0024 (9)	0.0032 (9)
C14	0.0225 (12)	0.061 (2)	0.0390 (14)	−0.0028 (12)	−0.0078 (10)	0.0057 (13)
C15	0.0289 (13)	0.0446 (17)	0.0448 (14)	0.0154 (12)	−0.0089 (11)	−0.0008 (12)
N1	0.0226 (9)	0.0215 (10)	0.0252 (9)	−0.0005 (8)	−0.0012 (7)	0.0009 (7)
N2	0.0184 (9)	0.0235 (10)	0.0228 (8)	0.0015 (7)	−0.0003 (7)	0.0018 (7)
N3	0.0183 (9)	0.0211 (10)	0.0299 (9)	0.0039 (8)	−0.0050 (7)	0.0009 (7)
N4	0.0207 (10)	0.0404 (13)	0.0371 (10)	0.0009 (9)	−0.0056 (8)	0.0058 (9)
O1	0.0233 (8)	0.0224 (9)	0.0424 (9)	0.0016 (7)	−0.0081 (7)	0.0008 (7)
Cl1	0.0256 (3)	0.0239 (3)	0.0319 (3)	0.0018 (2)	−0.0017 (2)	−0.0059 (2)
Cl2	0.0302 (3)	0.0234 (3)	0.0321 (3)	0.0013 (2)	0.0022 (2)	0.0035 (2)
Zn1	0.02174 (15)	0.01823 (15)	0.02718 (15)	0.00072 (10)	−0.00272 (10)	−0.00081 (9)

### Geometric parameters (Å, º)

**Table:**

C1—N1	1.335 (3)	C10—H10A	0.9300
C1—C2	1.391 (3)	C11—N4	1.360 (3)
C1—H1A	0.9300	C11—C12	1.404 (3)
C2—C3	1.373 (4)	C12—C13	1.375 (3)
C2—H2A	0.9300	C12—H12A	0.9300
C3—C4	1.392 (3)	C13—H13A	0.9300
C3—H3A	0.9300	C14—N4	1.452 (3)
C4—C5	1.380 (3)	C14—H14A	0.9600
C4—H4A	0.9300	C14—H14B	0.9600
C5—N1	1.348 (3)	C14—H14C	0.9600
C5—C6	1.470 (3)	C15—N4	1.453 (3)
C6—N2	1.280 (3)	C15—H15A	0.9600
C6—H6A	0.9300	C15—H15B	0.9600
C7—O1	1.237 (3)	C15—H15C	0.9600
C7—N3	1.384 (3)	N1—Zn1	2.2080 (18)
C7—C8	1.456 (3)	N2—N3	1.358 (2)
C8—C13	1.402 (3)	N2—Zn1	2.1122 (19)
C8—C9	1.403 (3)	N3—H3B	0.8600
C9—C10	1.369 (3)	O1—Zn1	2.2019 (15)
C9—H9A	0.9300	Cl1—Zn1	2.2282 (6)
C10—C11	1.419 (3)	Cl2—Zn1	2.2590 (6)
			
N1—C1—C2	123.0 (2)	C12—C13—H13A	119.2
N1—C1—H1A	118.5	C8—C13—H13A	119.2
C2—C1—H1A	118.5	N4—C14—H14A	109.5
C3—C2—C1	118.4 (2)	N4—C14—H14B	109.5
C3—C2—H2A	120.8	H14A—C14—H14B	109.5
C1—C2—H2A	120.8	N4—C14—H14C	109.5
C2—C3—C4	119.5 (2)	H14A—C14—H14C	109.5
C2—C3—H3A	120.2	H14B—C14—H14C	109.5
C4—C3—H3A	120.2	N4—C15—H15A	109.5
C5—C4—C3	118.5 (2)	N4—C15—H15B	109.5
C5—C4—H4A	120.8	H15A—C15—H15B	109.5
C3—C4—H4A	120.8	N4—C15—H15C	109.5
N1—C5—C4	122.6 (2)	H15A—C15—H15C	109.5
N1—C5—C6	114.65 (18)	H15B—C15—H15C	109.5
C4—C5—C6	122.8 (2)	C1—N1—C5	118.04 (19)
N2—C6—C5	115.7 (2)	C1—N1—Zn1	126.75 (16)
N2—C6—H6A	122.2	C5—N1—Zn1	115.20 (14)
C5—C6—H6A	122.2	C6—N2—N3	122.18 (19)
O1—C7—N3	118.41 (19)	C6—N2—Zn1	120.75 (15)
O1—C7—C8	123.9 (2)	N3—N2—Zn1	117.03 (14)
N3—C7—C8	117.7 (2)	N2—N3—C7	115.11 (18)
C13—C8—C9	117.7 (2)	N2—N3—H3B	122.4
C13—C8—C7	118.2 (2)	C7—N3—H3B	122.4
C9—C8—C7	124.1 (2)	C11—N4—C14	120.9 (2)
C10—C9—C8	121.0 (2)	C11—N4—C15	120.5 (2)
C10—C9—H9A	119.5	C14—N4—C15	118.4 (2)
C8—C9—H9A	119.5	C7—O1—Zn1	116.88 (14)
C9—C10—C11	121.5 (2)	N2—Zn1—O1	72.54 (6)
C9—C10—H10A	119.3	N2—Zn1—N1	73.56 (7)
C11—C10—H10A	119.3	O1—Zn1—N1	146.03 (7)
N4—C11—C12	121.6 (2)	N2—Zn1—Cl1	126.09 (5)
N4—C11—C10	121.2 (2)	O1—Zn1—Cl1	99.73 (5)
C12—C11—C10	117.2 (2)	N1—Zn1—Cl1	98.25 (5)
C13—C12—C11	120.9 (2)	N2—Zn1—Cl2	116.52 (5)
C13—C12—H12A	119.5	O1—Zn1—Cl2	97.90 (5)
C11—C12—H12A	119.5	N1—Zn1—Cl2	99.04 (5)
C12—C13—C8	121.6 (2)	Cl1—Zn1—Cl2	117.39 (2)

### Hydrogen-bond geometry (Å, º)

**Table:**

*D*—H···*A*	*D*—H	H···*A*	*D*···*A*	*D*—H···*A*
N3—H3*B*···Cl2^i^	0.86	2.49	3.199 (2)	140

Symmetry code: (i) *x*, −*y*+3/2, *z*+1/2.
